# Colonic Perforation in Critically Ill COVID-19 Patients: A Case Series Highlighting Surgical Management and Outcomes

**DOI:** 10.7759/cureus.70839

**Published:** 2024-10-04

**Authors:** Jeffrey John, Dileep Anto, Shameel Musthafa, Omar Moustafa, Ahmad Zarour

**Affiliations:** 1 Acute Care Surgery, Hamad Medical Corporation, Doha, QAT; 2 Surgery, Hamad Medical Corporation, Doha, QAT

**Keywords:** acute care surgery, colonic perforation, covid-19, covid-19 complication, emergency abdominal surgery, gastrointestinal perforation, global pandemic, large bowel perforation, severe acute respiratory infection, urgent laparotomy

## Abstract

The COVID-19 pandemic has primarily been characterized by respiratory symptoms, but emerging evidence suggests multi-organ involvement, including the gastrointestinal tract. This case report aims to highlight colon perforation as a rare but significant complication in COVID-19 patients.

We present two cases of male patients, aged 38 and 33, who were admitted with severe COVID-19 symptoms and required intubation and ventilator support. Both patients developed abdominal distension during their ICU stay, prompting surgical consultations.

In the first case, a 38-year-old male developed septic shock and abdominal distension on day 29 of his hospital stay. An exploratory laparotomy revealed a 2 mm perforation in the sigmoid colon, which was managed with Hartmann’s procedure. In the second case, a 33-year-old male developed abdominal distension one week post-admission. An exploratory laparotomy revealed a 2 mm perforation in the mid-transverse colon, managed initially with a primary two-layer repair and later with a transverse loop colostomy due to additional perforations.

These cases emphasize the importance of maintaining a high index of suspicion for gastrointestinal complications, such as colon perforation, in COVID-19 patients. Early diagnosis and surgical intervention are crucial for managing this life-threatening complication. Further research is needed to understand the incidence and underlying mechanisms of colon perforation in COVID-19 patients.

## Introduction

The COVID-19 pandemic, caused by the SARS-CoV-2, has had a profound impact on healthcare systems globally. Initially identified as a respiratory illness, COVID-19 has since been found to affect multiple organ systems, including the GI tract. While respiratory symptoms such as cough, fever, and shortness of breath are the most commonly observed manifestations, a growing body of evidence suggests that GI symptoms like diarrhea, abdominal pain, and vomiting are also prevalent among COVID-19 patients [1].

The significance of GI involvement in COVID-19 extends beyond mere symptomatology. Emerging reports indicate that complications such as colon perforation, although rare, can occur in COVID-19 patients and may lead to severe outcomes if not promptly addressed. De Nardi P et al. reported the first case of colonic perforation in a 53-year-old male patient who was in the ICU, intubated, and on mechanical ventilation. This patient had been diagnosed with COVID-19 ten days before the perforation of the ascending colon [1]. Colon perforation is a medical emergency characterized by a rupture or hole in the colon, resulting in the leakage of intestinal contents into the abdominal cavity. This can lead to severe infection, sepsis, and even death. Given the life-threatening nature of this complication, timely diagnosis and surgical intervention are often required [2].

The incidence of colon perforation in COVID-19 patients is not yet well-established, making it a critical area for clinical awareness and research. This case report aims to contribute to the existing literature by presenting two cases of colon perforation in COVID-19 patients. The objective is threefold: first, to add to the growing body of evidence regarding the extrapulmonary manifestations of COVID-19; second, to emphasize the importance of early recognition and diagnosis for timely surgical intervention; and third, to encourage further research into understanding the underlying mechanisms and risk factors for colon perforation in COVID-19 patients.

In summary, these case reports serve as a crucial reminder for clinicians to maintain a high index of suspicion for colon perforation when treating COVID-19 patients presenting with GI symptoms. It aims to shed light on this rare but significant complication, thereby informing clinical practice and guiding future research.

## Case presentation

Case 1

Patient Information and Background

A 38-year-old male patient was admitted under internal medicine with 3-4 days of fever, cough, and shortness of breath for 1 day. He tested positive for COVID-19, severe variety, and required admission to the ICU, intubation, and ventilator support on the same day of admission. He was treated with Azithromycin, Lopinavir, Hydroxychloroquine, Enoxaparin, Ceftriaxone, and a proton pump inhibitor. He has no history of smoking, alcohol, or drug abuse, no past medical diseases, malignancies, or prior operations, and no relevant family history of gastrointestinal or hematological diseases.

Presentation and Clinical Findings

Ten days later, he was extubated but desaturated within 24 hours and had to be re-intubated. On day 29 of his hospital stay, he developed features of septic shock with unstable vital signs such as fever, tachycardia, hypotension, tachypnea, and desaturation, along with a sudden onset of abdominal distension while in the ICU, prompting a surgical consult. Relevant history could not be elicited due to his intubated status. During clinical examination, he was hypotensive on vasopressor support, and evaluation of tenderness, rebound, or guarding was limited. His abdomen was soft but distended with no visible peristalsis or pulsations, localized erythema, or scars. No masses or organomegaly were palpable. Percussion revealed a tympanic note even over the liver, denoting entrapped gas. Bowel sounds were absent on auscultation.

Diagnostic Assessment

**Figure 1:**
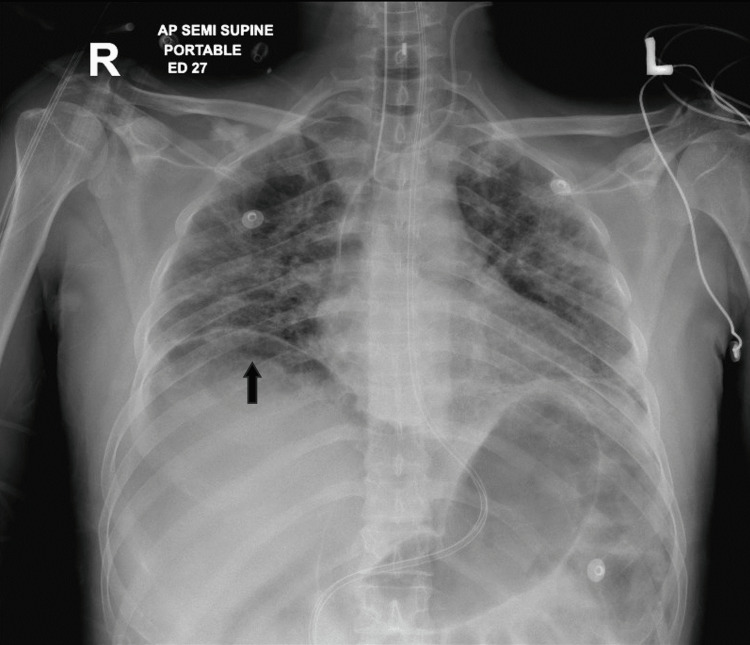
X-ray of the chest and upper abdomen in a semi-sitting position. The black arrow points to the free air under the right diaphragm, suggestive of bowel perforation.

**Figure 2:**
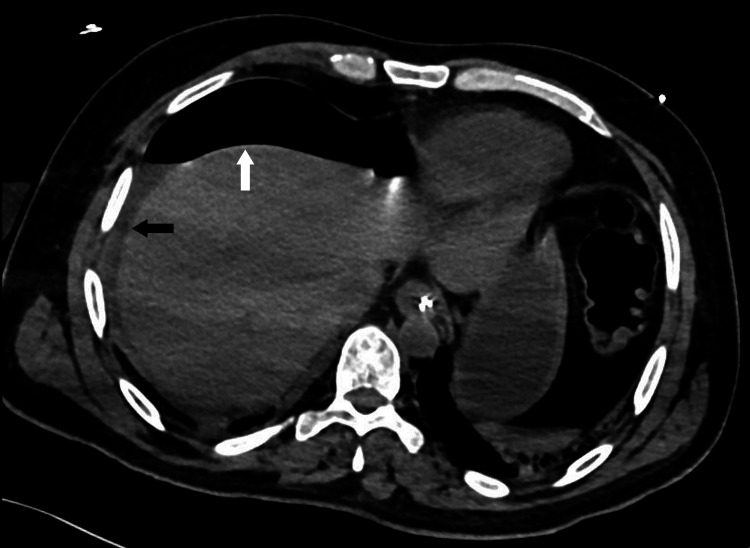
CT scan of the abdomen showing pneumoperitoneum. The white arrow points to pneumoperitoneum anterior to the liver. The black arrow indicates free fluid in the peritoneal cavity.

X-ray of the chest showed a large amount of free air under the right diaphragm and in the peritoneal cavity (Figure 1). A CT scan of the abdomen and pelvis with IV contrast confirmed a large volume of pneumoperitoneum in the prehepatic area extending down to the mid-abdomen and a moderate amount of free fluid in the perihepatic region (Figure 2), as well as in the pelvis with multiple small air locules seen within the pelvic free fluid. No definite bowel wall defect or discontinuity could be visualized. There was marked distension of the sigmoid colon loaded with feces with no definite distal obstructing pathology. There was mild thickening of the ascending colon with abnormal enhancement and some reactive inflammatory changes with no evidence of bowel obstruction.

Surgical Intervention

A prompt emergency exploratory laparotomy under antibiotic coverage with complete COVID precaution protocols revealed a 2 mm perforation in the sigmoid colon with fecal peritonitis. We performed a thorough lavage with saline and a Hartmann’s procedure using a 100 mm mechanical stapler for the distal stump, which was reinforced with a PDS suture. The end stoma was created in the left iliac fossa. Surgical drains were placed in the left paracolic gutter and the pelvis. Histopathological examination of the resected specimen of sigmoid demonstrated a wall defect with transmural inflammation comprising neutrophils, hemorrhage, and edema. The surgical margins were viable but involved in inflammation.

Postoperative Care

Total parenteral nutrition was initiated on the 3rd postoperative day (POD), and oral fluids on the 4th POD. Surgical drains were removed on the 10th POD, and sutures were removed in the 3rd week after surgery. He continued under the care of the medical team until he recovered from COVID-19 and was transferred to the long-term care unit for rehabilitation and management of pressure ulcers, which he developed during long ICU care. After a prolonged hospital stay, with physiotherapy and wound care support for his COVID-19-related sequelae and pressure ulcer, he was discharged.

Follow-up and Outcomes

Six months later, he underwent a colonoscopy, which showed normal mucosa and no pathology, followed by a reversal of Hartmann’s procedure with re-anastomosis of the colon and was discharged in good health.

Case 2

Patient Information and Background

A 33-year-old male patient of Southeast Asian ethnicity was admitted under medical care with symptoms of fever, headache, body aches, sore throat, cough for three days, and shortness of breath for 1 day. He tested positive for COVID-19 and was admitted to the medical unit. Due to desaturation, he was intubated and ventilated the following day. His treatment regimen included Azithromycin, Hydroxychloroquine, Oseltamivir, Enoxaparin, and a single dose of Tocilizumab. He had no history of smoking, alcohol, or drug abuse. He also had no past medical diseases, malignancies, or prior operations, and no relevant family history of GI or hematological diseases.

Presentation and Clinical Findings

One week post-admission, the patient developed abdominal distension while in the ICU. A CT scan of the abdomen was performed, revealing large bowel dilatation from the cecum to the rectum with no evidence of obstruction, ascitic fluid, or free intraperitoneal air. The small bowel appeared unremarkable, and mesenteric vessels were patent.

Diagnostic Assessment

**Figure 3:**
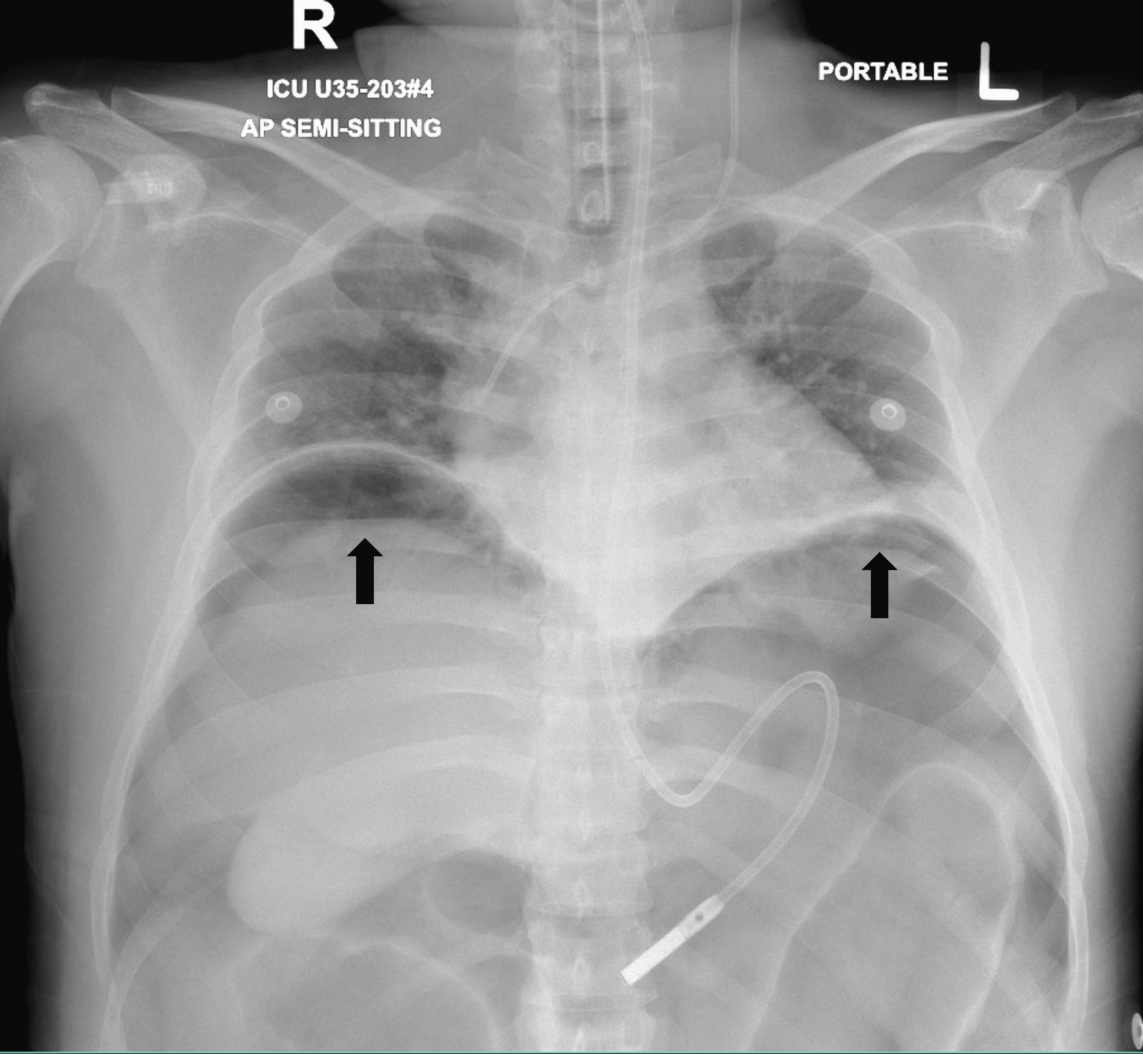
X-ray of the chest and upper abdomen in a semi-sitting position. Black arrows show air under both hemi-diaphragms.

A surgical consultation was sought the next day due to persistent abdominal distension. The patient was intubated but not sedated or paralyzed. His abdomen was distended and tense, with an intra-abdominal pressure (IAP) of 30 mmHg. No organomegaly or masses were palpable. Exaggerated bowel sounds were noted, and a digital rectal examination revealed an empty, roomy rectum. A follow-up X-ray done the next day showed pneumoperitoneum suggestive of bowel perforation (Figure 3).

Treatment Intervention and Postoperative Care

**Figure 4:**
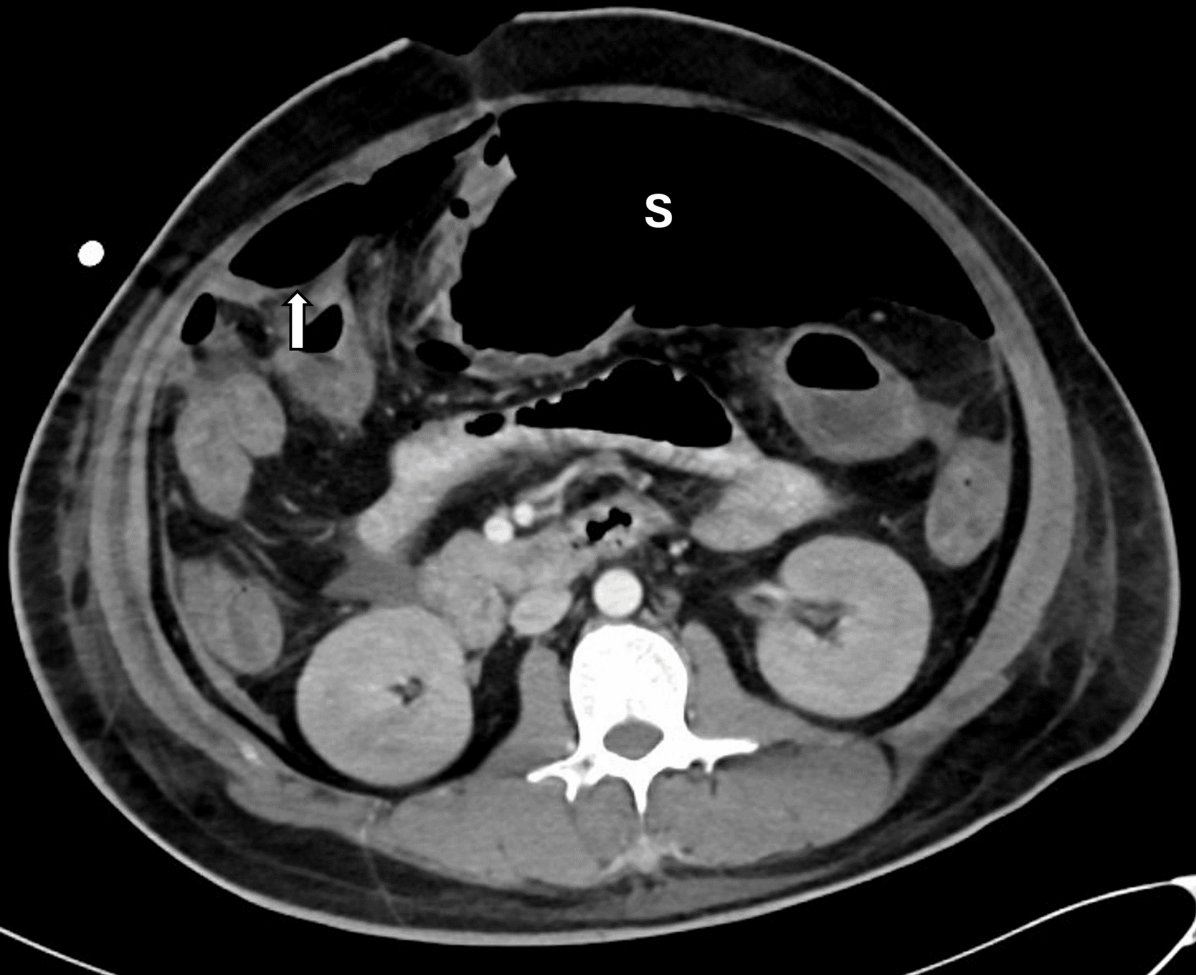
CT scan of the abdomen with IV contrast. The white arrow points to pneumoperitoneum anterior to the liver, which is more than the expected level of free air for a post-laparotomy patient on postoperative day (POD) 9. The letter 'S' (in white) indicates the over-distended stomach.

An emergency exploratory laparotomy revealed a 2 mm perforation in the mid-transverse colon and fecal peritonitis. A primary two-layer repair was performed along with thorough lavage. Four days postoperatively, the patient passed stool, and nasojejunal tube feeding was started. However, he developed renal failure, requiring dialysis. On the 9th POD, he again showed abdominal distension and leukocytosis. Further investigations, including an X-ray, showed free air under the diaphragm, and a CT scan demonstrated gastric over-distension associated with significant free intraperitoneal air as well as free ascitic fluid (Figure 4). There was extraluminal contrast fluid layering at the recto-vesical pouch raising suspicion of another bowel perforation.

An emergency re-exploratory laparotomy was performed, revealing two additional perforations adjacent to the previous one; one on the antimesenteric and the other on the mesenteric border of the transverse colon. A transverse loop colostomy was performed, leading to a smooth recovery. Ten days later, he was transferred out of the ICU to the medical ward to manage pleural effusion and COVID-related issues and was discharged three weeks later.

Follow-up and Outcomes

The patient remained under follow-up for three months and was doing well. A reversal of the colostomy was advised but the patient opted to have the procedure done in his home country.

## Discussion

Colon perforation is an increasingly recognized but rare complication in patients with severe COVID-19, often associated with significant morbidity and mortality. Masanam MK et al. noted the increased incidence of severe colonic perforations during the COVID-19 pandemic, suggesting that COVID-19-related colonic perforations, though rare, are becoming more frequently recognized [3]. This highlights the critical need for monitoring GI symptoms in critically ill COVID-19 patients.

This report presents two cases of colon perforation in young male patients (aged 38 and 33) with severe COVID-19 who required ICU admission and mechanical ventilation. Both developed abdominal distension during their ICU stay, necessitating surgical intervention. Compared to previously reported cases in the literature (Table 1), where the median age was 60 years, our patients were notably younger. The male predominance observed in our cases aligns with broader data showing that 69.4% of reported cases involve male patients.

**Table 1:** Compilation of published cases of large bowel perforations in COVID-19 patients (up to March 2023, in chronological order of reporting). Dx: Diagnosis; MV: Mechanically ventilated; M: Male; F: Female; Y: Yes; N: No; NR: Not recorded.

Author, Year	Month, Year	Country	Age	Sex	Days of COVID-19 Dx	ICU stay	MV	Part of colon	Management	Outcome
1. De Nardi P et al. [1]	May, 2020	Italy	53	M	10	Y	Y	Ascending Colon	Right colectomy + ileo-transverse anastomosis	Discharged
2. Corrêa Neto IJ et al. [4]	May, 2020	Brazil	80	F	0	Y	Y	Sigmoid	Recto-sigmoidectomy + terminal colostomy	Died
3. Almeida Vargas A et al. [5]	July, 2020	Spain	76	M	NR	Y	Y	Ischemic colitis	Not operated	Died
			68	M	NR	Y	Y	Cecum	Ileostomy	Died
			56	M	NR	Y	Y	Colon	Not operated	Died
4. Rojo M et al. [6]	July, 2020	Spain	54	F	15	Y	Y	Cecum	Right hemicolectomy	Died
5. Nahas SC et al. [7]	July, 2020	Brazil	92	M	0	N	N	Descending colon	Resection + terminal colostomy	Died
6. Persiano T et al. [8]	September, 2020	Italy	73	M	11	N	N	Ascending colon	Right hemicolectomy	Discharged
7. Giuffrè M et al. [9]	October, 2020	Italy	87	F	0	N	N	Rectum	Not operated	Died
8. Parhar G et al. [10]	October, 2020	USA	36	F	NR	Y	Y	Cecum	Right hemicolectomy + loop ileostomy	Died
9. Verma D et al. [11]	December, 2020	India	60	F	11	N	NR	Rectum	Transverse colostomy	Discharged
10. Bulte JP et al. [12]	February, 2021	Netherlands	65	M	28	Y	Y	Rectum	Diversion colostomy	Discharged
			58	M	14	Y	Y	Cecum	Ileocecectomy + end ileostomy	Discharged
			57	M	14	Y	Y	Transverse colon	Extended right hemicolectomy + end ileostomy	Discharged
11. Estevez-Cerda SC et al. [13]	March, 2021	Mexico	34	M	07	Y	Y	Ascending colon	Right colectomy	Discharged
			54	M	08	Y	Y	Ascending colon	Right colectomy	Discharged
			69	M	16	Y	Y	Ascending colon	Right colectomy	Died
			60	F	12	Y	Y	Sigmoid + Terminal ileum	Hartmann’s resection + end to end anastomosis	Discharged
12. Al Argan RJ et al. [14]	May, 2021	Saudi Arabia	70	M	44	Y	Y	Cecum	Not operated	Discharged
			37	M	05	Y	Y	Ascending colon	Not operated	Discharged
			74	M	04	N	N	Sigmoid	Hartman’s resection + colostomy	Discharged
13. Nakatsutsumi K et al. [15]	August, 2021	Japan	67	M	12	Y	Y	Transverse Colon	Resection & colostomy	Died
14. Almeida A et al. [16]	September, 2021	Spain	79	M	NR	Y	Y	Sigmoid	Hartman’s resection	Discharged
15. Muñoz CA et al. [2]	September, 2021	Columbia	50	M	15	Y	Y	Hepatic Flexure	Primary closure & lavage	Discharged
16. Morimoto Y et al. [17]	January, 2022	Japan	79	M	08	Y	Y	Sigmoid	Transverse colostomy	Discharged
17. Chaugale SB et al. [18]	April, 2022	India	68	F	16	Y	NR	Sigmoid	Not operated	Discharged
			57	F	19	Y	NR	Cecum	Percutaneous drainage	Discharged
			72	M	18	N	NR	Cecum	Percutaneous drainage	Discharged
			25	M	22	N	NR	Ascending colon	Percutaneous drainage	Discharged
			56	M	24	Y	NR	Ascending colon	Repair of perforation + loop ileostomy	Died
			71	M	17	N	NR	Sigmoid	Not operated	Died
18. Masanam MK et al. [3]	August, 2022	USA	82	F	14	Y	Y	Ascending colon	Right hemicolectomy + end ileostomy	Died
			45	M	13	Y	Y	Right Colon	Ileocecectomy+ end ileostomy	Discharged
			51	M	21	Y	Y	Cecum	Right Hemicolectomy	Died
19. Guiritan AT and Cataluña JG [19]	March, 2023	Philippines	35	F	07	Y	Y	Cecum	Right hemicolectomy	Died

The pathophysiology behind colonic perforations in COVID-19 remains multifactorial, involving direct viral invasion, a cytokine storm, and ischemic injury to the bowel. Keshavarz P et al. discussed the direct invasion of SARS-CoV-2 into the GI tract, suggesting that viral-induced mucosal damage and inflammatory response may contribute to complications such as bowel perforation [20]. Understanding these mechanisms is critical, as it supports the need for aggressive diagnostic and therapeutic approaches when faced with GI manifestations in COVID-19 patients. These mechanisms underscore the need for a high level of clinical vigilance and a tailored approach to managing these high-risk patients.

Al Argan RJ et al. emphasized the importance of high clinical suspicion and timely surgical intervention in managing GI complications in COVID-19 patients [14]. Similarly, Muñoz CA et al. underscored the necessity of a prompt surgical response to prevent septic complications in large intestinal perforations secondary to COVID-19 [2]. Both patients in our series underwent emergency laparotomies with differing surgical approaches: one underwent Hartmann’s procedure, and the other had a primary repair followed by a loop colostomy during re-exploratory surgery. This variability in surgical intervention is consistent with reported cases, where management strategies have ranged from conservative approaches to more invasive procedures like ileostomy and primary repair.

Despite the critical presentation and need for extensive surgical intervention, both of our patients were eventually discharged, consistent with the 58.3% discharge rate observed in previously reported cases. However, the overall mortality rate of 41.7% in the broader literature underscores the high-risk nature of these conditions, highlighting the comparatively favorable outcomes for our cases, possibly due to their younger age and fewer comorbidities. This suggests that while the overall trends in ICU and ventilator requirements, as well as outcomes, were similar, our patients’ younger age and lack of prior medical conditions may have contributed to their more favorable prognosis despite the severe nature of their clinical course.

## Conclusions

In conclusion, comparing our cases of colon perforation in COVID-19 patients with published case reports and case series provides valuable insights into the presentation, surgical treatment, and outcomes of this rare complication. The literature suggests that prompt recognition, diagnosis, and surgical intervention are crucial for managing colon perforation in COVID-19 patients. Further research is needed to better understand the incidence, risk factors, and optimal management strategies for colon perforation in COVID-19 patients.
